# neoANT-HILL: an integrated tool for identification of potential neoantigens

**DOI:** 10.1186/s12920-020-0694-1

**Published:** 2020-02-22

**Authors:** Ana Carolina M. F. Coelho, André L. Fonseca, Danilo L. Martins, Paulo B. R. Lins, Lucas M. da Cunha, Sandro J. de Souza

**Affiliations:** 1 Bioinformatics Multidisciplinary Enviroment (BioME), Institute Metropolis Digital, Federal University of Rio Grande do Norte, UFRN, Natal, Brazil; 2 PhD Program in Bioinformatics, UFRN, Natal, Brazil; 3 Brain Institute, Federal University of Rio Grande do Norte, UFRN, Natal, Brazil; 4 Institutes for Systems Genetics, West China Hospital, Sichuan University, Chengdu, China

**Keywords:** Neoantigens, Cancer, Immunogenomic analyses

## Abstract

**Background:**

Cancer neoantigens have attracted great interest in immunotherapy due to their capacity to elicit antitumoral responses. These molecules arise from somatic mutations in cancer cells, resulting in alterations on the original protein. Neoantigens identification remains a challenging task due largely to a high rate of false-positives.

**Results:**

We have developed an efficient and automated pipeline for the identification of potential neoantigens. neoANT-HILL integrates several immunogenomic analyses to improve neoantigen detection from Next Generation Sequence (NGS) data. The pipeline has been compiled in a pre-built Docker image such that minimal computational background is required for download and setup. NeoANT-HILL was applied in The Cancer Genome Atlas (TCGA) melanoma dataset and found several putative neoantigens including ones derived from the recurrent RAC1:P29S and SERPINB3:E250K mutations. neoANT-HILL was also used to identify potential neoantigens in RNA-Seq data with a high sensitivity and specificity.

**Conclusion:**

neoANT-HILL is a user-friendly tool with a graphical interface that performs neoantigens prediction efficiently. neoANT-HILL is able to process multiple samples, provides several binding predictors, enables quantification of tumor-infiltrating immune cells and considers RNA-Seq data for identifying potential neoantigens. The software is available through github at https://github.com/neoanthill/neoANT-HILL.

## Background

Recent studies have demonstrated that T cells can recognize tumor-specific antigens that bind to human leukocyte antigens (HLA) molecules at the surface of tumor cells [1, 2]. During tumor progression, accumulating somatic mutations in the tumor genome can affect protein-coding genes and result in mutated peptides [1]. These mutated peptides, which are present in the malignant cells but not in the normal cells, may act as neoantigens and trigger T-cell responses due to the lack of thymic elimination of autoreactive T-cells (central tolerance) [3–5]. As result, these neoantigens appear to represent ideal targets attracting great interest for cancer immunotherapeutic strategies, including therapeutic vaccines and engineered T cells [1, 6].

In the last few years, advances in next-generation sequencing have provided an accessible way to generate patient-specific data, which allows the prediction of tumor neoantigens in a rapid and comprehensive manner [7]. Several approaches have been developed, such as pVAC-Seq [8], MuPeXI [9], TIminer [10] and TSNAD [11], which predict potential neoantigens produced by non- synonymous mutations. However, none of these proposed tools considers tumor transcriptome sequencing data (RNA-seq) for identifying somatic mutations. Moreover, only one of these tools provides quantification of the fraction of tumor-infiltrating immune cell types (Supplementary: Table S1).

**Fig. 1:**
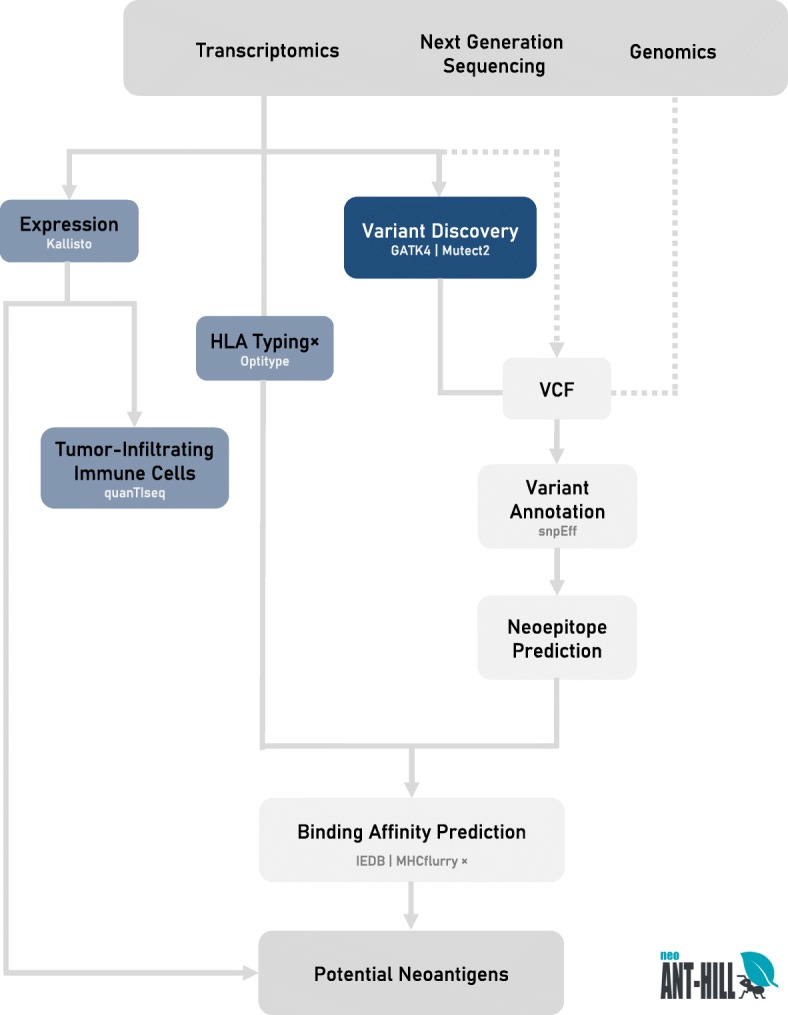
Overall workflow of neoANT-HILL. The neoANT-HILL was designed to analyze NGS data, such as genome (WGS or WES) and transcriptome (RNA-Seq) data. Basically, it takes as input distinct data types, including raw and pre-aligned sequences from RNA-Seq, as well as, variant calling files (VCF) from genome or transcriptome data (dotted lines indicate that the VCF must be previously created by the user). The blue boxes represent the transcriptome analyses, which should be carried out using data in either BAM format (variant calling) or fastq format (expression, HLA typing and tumor-infiltrating immune cells). The neoANT-HILL can perform gene expression (Kallisto), variant calling (GATK4 | Mutect2), HLA typing (Optitype), and Tumor-infiltrating immune cells (quanTIseq). The gene expression quantification is used as input to identify molecular signatures associated with immune cell diversity into the tumor samples. On the other hand, the gray boxes represent common steps to genome and transcriptome data. NeoANT-HILL uses the variant calling data to reconstruct the proteins sequences using as reference the NCBI RefSeq database. The VCF files can be either generated by using our pipeline or by external somatic variant-calling software. Next, reconstructed proteins are submitted to neoepitope binding prediction using HLA alleles from Optitype results or defined by the user. Finally, all steps and results are shown into a user-friendly interface

Here we present a versatile tool with a graphical user interface (GUI), called neoANT-HILL, designed to identify potential neoantigens arising from cancer somatic mutations. neoANT-HILL integrates complementary features to prioritizing mutant peptides based on predicted binding affinity and mRNA expression level (Fig. 1). We used datasets from GEUVADIS RNA sequencing project [12] to demonstrate that RNA-seq is also a potential source of mutation detection. Finally, we applied our pipeline on a large melanoma cohort from The Cancer Genome Atlas [13] to demonstrate its utility in predicting and suggesting potential neoantigens that could be used in personalized immunotherapy.

### Implementation

neoANT-HILL requires a variant list for potential neoantigen prediction. Our pipeline is able to handle a VCF file (single- or multi-sample) for the genome data or a tumor transcriptome sequence data (RNA-seq) in which somatic mutation will be called following GATK best practices [14, 15] with Mutect2 [16] on tumor-only mode. However, the RNA-seq data must be previously aligned to the reference genome (BAM) by the user. The size of corresponding BAM files from the RNA-Seq can be a limiting factor in the analysis. Since neoANT-HILL is run locally, the user must guarantee that enough space and memory are available for a proper execution of the program. In the current implementation, neoANT-HILL supports VCF files generated using the human genome version GRCh37. The variants are properly annotated by snpEff [17] to identify non-synonymous mutations (missense, frameshift and inframe).

Once the VCF files have been annotated, the resulting altered amino acid sequences are inferred from the NCBI Reference Sequence database (RefSeq) [18]. For frameshift mutations, the altered amino acid sequence is inferred by translating the resulting cDNA sequence. Altered epitopes (neoepitopes) are translated into a 21-mer sequence where the altered residue is at the center. If the mutation is at the beginning or at the end of the transcript, the neoepitope sequence is built by taking the 20 following or preceding amino acids, respectively. The neoepitope sequence and its corresponding wild-type are stored in a FASTA file. Non-overlapping neoepitopes can be derived from frameshift mutations.

A list of HLA haplotypes is also required. If this data had not been provided by the user, neoANT-HILL includes the Optitype algorithm [19] to infers class-I HLA molecules from RNA-Seq. The subsequent step is the binding affinity prediction between the predicted neoepitopes and HLA molecules. This can be executed on single or multi-sample using parallelization with the custom configured parameters. The correspondent wild-type sequences are also submitted at this stage, which allows calculation of the fold change between wild-type and neoepitopes binding scores, known as differential agretopicity index (DAI) as proposed by [20]. This additional neoantigen quality metric contributes to a better prediction of neoantigens that can elicit an antitumor response [21].

neoANT-HILL employs seven binding prediction algorithms from Immune Epitope Database (IEDB) [22], including NetMHC (v. 4.0) [23, 24], NetMHCpan (v. 4.0) [25], NetMHCcons [26], NetMHCstabpan [27], PickPocket [28], SMM [29] and SMMPMBEC [30], and the MHCflurry algorithm [31] for HLA class I. The user is able to specify the neoepitope lengths to perform binding predictions. Each neoepitope sequence is parsed through a sliding window metric. Our pipeline also employs four IEDB-algorithms for HLA class II binding affinity prediction: NetMHCIIpan (v. 3.1) [32], NN-align [33], SMM- align [34], and Sturniolo [35].

Moreover, when the unmapped RNA-seq reads are available (fastq), neoANT-HILL can quantify the expression levels of genes carrying a potential neoantigen. Our pipeline uses the Kallisto algorithm [36] and the output is reported as transcripts per million (TPM). Potential neoantigens arising from genes showing an expression level under 1 TPM are excluded. In addition, neoANT-HILL also offers the possibility of estimating quantitatively, via deconvolution, the relative fractions of tumor-infiltrating immune cell types through the use of quanTIseq [37].

Our software was developed under a pre-built Docker image. The required dependencies are packed up, which simplify the installation process and avoid possible incompatibilities between versions. As previously described, several analyses are supported and each one relies on different tools. Several scripts were implemented on Python to complete automate the execution of these single tools and data integration.

## Results

**Fig. 2:**
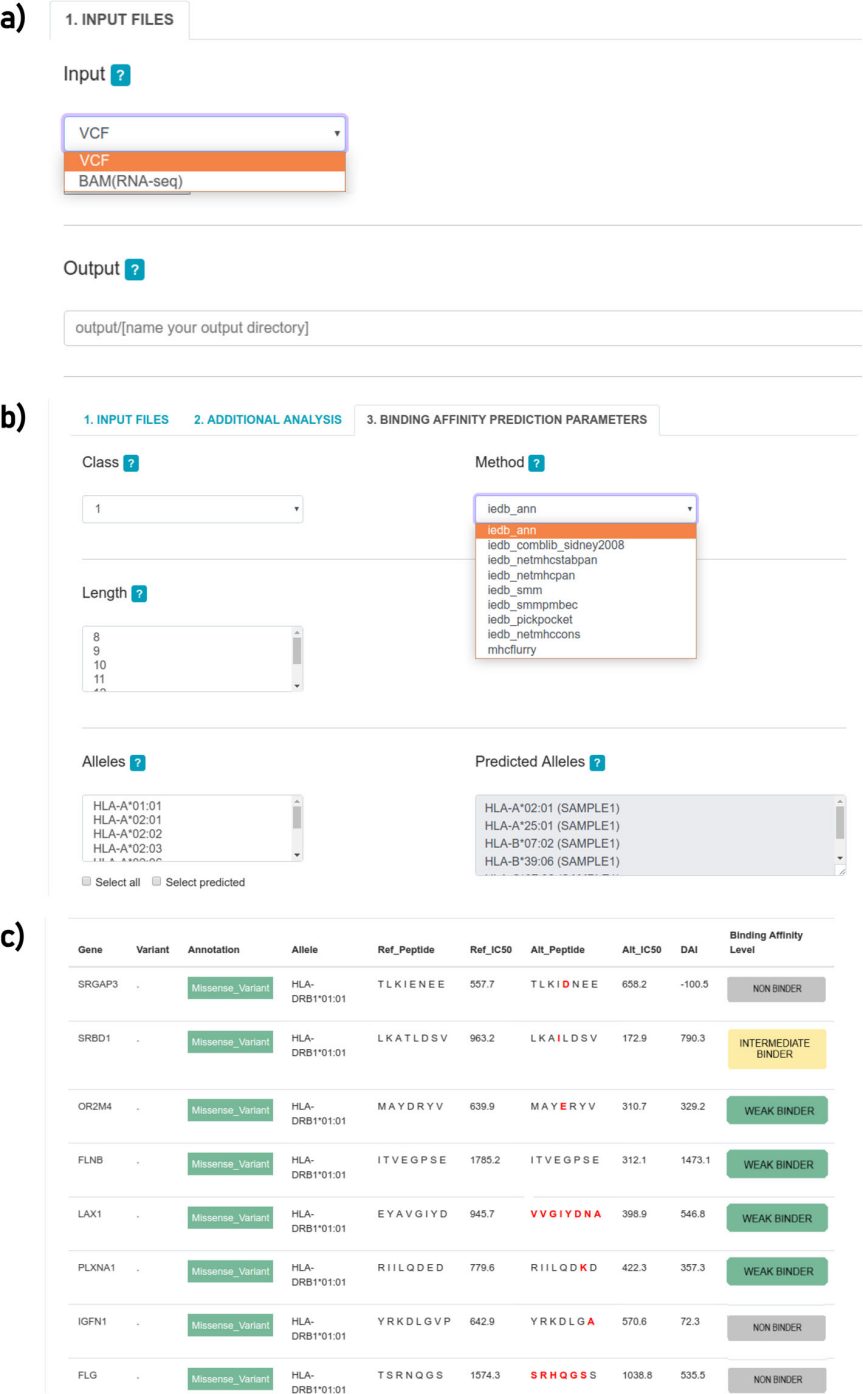
Screenshots of neoANT-HILL interface. **a** Processing tab for submitting genome or transcriptome data. **b** Processing tab for parameters selection to run neoepitope binding affinity prediction. On this tab, all the parameters can be defined by the users through selection boxes, ranging from the MHC class, corresponding prediction methods, to parallelization settings. The length and HLA alleles parameters allow multiple selections, although that might interfere in the processing time. **c** Binding prediction results tab shows an interactive table which reports all predicted neoepitopes and information about each prediction, respectively. The interactive table shows several columns, such as the donor gene, HLA allele, mutation type, reference (Ref_Peptide) and altered (Alt_peptide) peptides sequences, reference (Ref_IC50) and altered (Alt_IC50) binding affinity scores, binding affinity category (High, Moderate, Low, and Non-binding) and differential agretopicity index (DAI)

neoANT-HILL was designed through a user-friendly graphical interface (Fig. 2) implemented on Flask framework. The interface comprises three main sections: (i) Home (Fig. 2a), (ii) Processing (Fig. 2b), and (iii) Results (Fig. 2c). neoANT-HILL stores the outputs in sample-specific folders. Our pipeline provides a table of ranked predicted neoantigens with HLA alleles, variant information, binding prediction score (neoepitope and wild-type) and binding affinity classification. When optional analyses are set by the user, the outputs are stored in separated tabs. Gene expression is provided as a list with corresponding RNA expression levels and it is used to filter the neoantigens candidates.

### Variant identification on RNA-Seq

We evaluate the utility of RNA-seq for identifying frameshift, indels and point mutations by using samples (*n = 15*) from the GEUVADIS RNA sequencing project. Although these samples are not derived from tumor cells, the goal of these analysis was to benchmark the efficiency of our pipeline to detect somatic mutations from RNA-Seq data. We limited our analysis to variants with read depth (DP) > = 10 and supported by at least five reads. The overall called variants were then compared to the corresponding genotypes (same individuals) provided by the 1000 Genomes Project Consortium (1KG) [38]. We found that on average 71% of variants in coding regions detected by RNA-seq were confirmed by the genome sequencing (concordant calls) (Supplementary Table S2). Variants in genes that are not expressed cannot be detected by RNA-seq and RNA editing sites could partially explain the discordant calls. Furthermore, some of the discrepancies can be also due to low coverage in the genome sequence, which generated a false-negative in the calling. Although calling variants from RNA-Seq data has been shown to be more challenging, it is an interesting alternative for genome sequencing and a large amount of tumor RNA-seq samples do not have normal matched data [39, 40].

### Use case

We applied our pipeline on a large melanoma cohort (SKCM, *n* = 466) from TCGA to demonstrate its utility in identifying potential neoantigens. We found approximately 198,000 instances of predicted neoantigens binding to HLA-I. It is important to note that the large number of mutant peptides is due to: i) the larger cohort size, ii) the high mutational burden of melanoma and iii) the large set of HLA alleles that was used to run the binding prediction. These neoepitopes were classified as strong (IC50 under 50 nM), intermediate (IC50 between 50 nM and 250 nM) or weak binders (IC50 over 250 nM and under 500 nM) (Supplementary Table S3). We limited our analyses to high binding affinity candidates to reduce potential false positives.

**Fig. 3:**
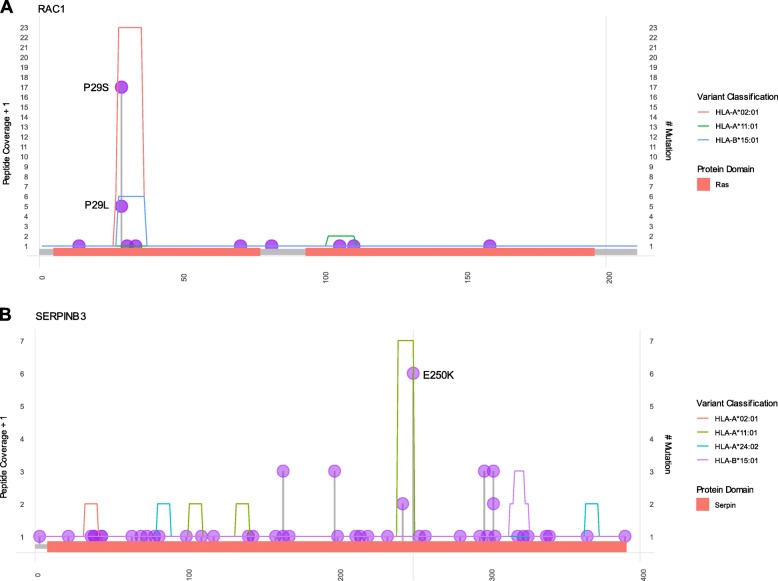
Distribution of recurrent missense mutations that generated high-affinity neoantigens. The y-axis shows peptide coverage based on the number of epitope binding predictions in each region. The coverage was calculated by increasing the overall frequency of each amino acid by one, including non-high-affinity regions. The allele classification is shown as colored lines. The x-axis shows the protein length, and also contains information about conserved domains for each protein. **a** P29S and RAC1 gene generated recurrent mutant peptides with strong affinity to HLA-A*02:01 and P29L generated peptides with strong affinity to HLA-A*02:01 or HLA-A*11:01, depending on peptide length (**b**) E250K in SERPINB3 gene generate a recurrent potential neoantigen that binds to HLA-A*11:01

We observed that the majority of strong binder mutant peptides are private and unique, which is likely linked to the high intratumor genetic diversity. However, we observed that frequent mutations may be likely to generate recurrent mutant peptides (Table 1). These recurrent neoantigens are interesting since they could be used as a vaccine for more than one patient. Figure 3 shows potential neoepitopes arising from recurrent mutations. The potential neoantigen (F**S**GEYIPTV), which was predicted to form a complex with HLA-A*02:01 allele, was found to be shared among 17 samples (3.65%). It was generated from the P29S mutation in gene RAC1 (Fig. 3a). RAC1 P29S have been described as a candidate biomarker for treatment with anti-PD1 or anti-PD-L1 antibodies [41]. Another mutation (P29L) in the same gene formed a recurrent potential neoantigen (F**L**GEYIPTV) and was found in 5 samples (1.07%). As another example, we can also highlight the potential shared neoantigen (LSMIVLLPN**K**) related to mutation E250K in the SERPINB3 gene (Fig. 3b). This was found in 6 samples (1.29%) and it was likely to form a complex with the HLA-A*11:01 allele. Mutations in SERPINB3 have also been related to response to immunotherapy [42].

**Table 1:** Top 15 potential shared neoantigens based on TCGA-SKCM cohort. Recurrent mutations observed on TCGA-SKCM cohort. The amino acid (AA) residue changes caused by somatic mutations are highlighted in the (neo) epitopes sequences. The frequency represents the number of samples showing the corresponding mutation

Gene	AA change	Neoepitope	HLA haplotype	Frequency
RAC1	P29S	FSGEYITV	HLA-A*02:01	17/466
KLHDC7A	E635K	HTATVRAKK	HLA-A*11:01	12/466
INMT	S212F	YMVGKREFFCV	HLA-A*02:01	9/466
CDH6	S524L	FLFSLAPEAA	HLA-A*02:01	8/466
ZBED2	E157K	GTMALWASQRK	HLA-A*11:01	8/466
CRNKL1	S128F	LQVPLPVPRF	HLA-A*15:01	7/466
IL37	S202L	FLFQPVCKA	HLA-A*02:01	7/466
SERPINB3	E250K	LSMIVLLPNK	HLA-A*11:01	6/466
DNAJC5B	E22K	STTGEALYK	HLA-A*11:01	6/466
MYO7B	E512K	MSIISLLDK	HLA-A*11:01	6/466
MORC1	E878K	IQNTYMVQYK	HLA-A*11:01	6/466
SCN7A	S445F	IEMKKRSPIF	HLA-A*15:01	6/466
PSG9	E404K	KISKSMTVK	HLA-A*11:01	6/466
RAC1	P29L	FLGEYIPTV	HLA-A*02:01	5/466
NUTF2	Q20K	SSFIQHYYK	HLA-A*11:01	5/466

## Conclusion

We present neoANT-HILL, a completely integrated, efficient and user-friendly software for predicting and screening potential neoantigens. We have shown that neoANT-HILL can predict neoantigen candidates, which can be targets for immunotherapies and predictive biomarkers for immune responses. Our pipeline is available through a user-friendly graphical interface which enables its usage by users without advanced programming skills. Furthermore, neoANT-HILL offers several binding prediction algorithms for both HLA classes and can process multiple samples in a single running. Unlike the majority of existing tools, our pipeline enables the quantification of tumor-infiltrating lymphocytes and considers RNA-Seq data for variant identification. The source code is available at https://github.com/neoanthill/neoANT-HILL.

## Availability and requirements

**Project name:** neoANT-HILL.

**Project home page:** https://github.com/neoanthill/neoANT-HILL

**Operating system(s):** Unix-based operating system, Mac OS, Windows.

**Programming language:** Python 2.7.

**Other requirements:** Docker.

**License:** Apache License 2.0.

**Any restrictions to use by non-academics:** None.

## Supplementary information

**Additional file 1.**

**Additional file 2.**

**Additional file 3.**

## Data Availability

The RNA-Seq dataset from Geuvadis RNA sequencing project analyzed during the current study are available in the ArrayExpress database (http://www.ebi.ac.uk/arrayexpress/) under the accession number E-GEUV-1. The corresponding genotyping data (Phase I) from each sample are available from the 1000 Genomes Project and was downloaded from the FTP site hosted at the EBI. ftp://ftp.1000genomes.ebi.ac.uk/vol1/ftp/phase1/data/ (ftp://ftp.1000genomes.ebi.ac.uk/vol1/ftp/phase1/data/NA12812/exome_alignment/NA12812.mapped.SOLID.bfast.CEU.exome.20110411.bam, ftp://ftp.1000genomes.ebi.ac.uk/vol1/ftp/phase1/data/NA12749/exome_alignment/NA12749.mapped.illumina.mosaik.CEU.exome.20110521.bam, ftp://ftp.1000genomes.ebi.ac.uk/vol1/ftp/phase1/data/NA20510/exome_alignment/NA20510.mapped.SOLID.bfast.TSI.exome.20110521.bam, ftp://ftp.1000genomes.ebi.ac.uk/vol1/ftp/phase1/data/NA19119/exome_alignment/NA19119.mapped.illumina.mosaik.YRI.exome.20110411.bam, ftp://ftp.1000genomes.ebi.ac.uk/vol1/ftp/phase1/data/NA19204/exome_alignment/NA19204.mapped.illumina.mosaik.YRI.exome.20110411.bam, ftp://ftp.1000genomes.ebi.ac.uk/vol1/ftp/phase1/data/NA18498/exome_alignment/NA18498.mapped.illumina.mosaik.YRI.exome.20110411.bam, ftp://ftp.1000genomes.ebi.ac.uk/vol1/ftp/phase1/data/NA12489/exome_alignment/NA12489.mapped.SOLID.bfast.CEU.exome.20110411.bam, ftp://ftp.1000genomes.ebi.ac.uk/vol1/ftp/phase1/data/NA20752/exome_alignment/NA20752.mapped.illumina.mosaik.TSI.exome.20110521.bam, ftp://ftp.1000genomes.ebi.ac.uk/vol1/ftp/phase1/data/NA18517/exome_alignment/NA18517.mapped.illumina.mosaik.YRI.exome.20110521.bam, ftp://ftp.1000genomes.ebi.ac.uk/vol1/ftp/phase1/data/NA11992/exome_alignment/NA11992.mapped.SOLID.bfast.CEU.exome.20110411.bam, ftp://ftp.1000genomes.ebi.ac.uk/vol1/ftp/phase1/data/NA19144/exome_alignment/NA19144.mapped.illumina.mosaik.YRI.exome.20110411.bam, ftp://ftp.1000genomes.ebi.ac.uk/vol1/ftp/phase1/data/NA20759/exome_alignment/NA20759.mapped.illumina.mosaik.TSI.exome.20110521.bam, ftp://ftp.1000genomes.ebi.ac.uk/vol1/ftp/phase1/data/NA19137/exome_alignment/NA19137.mapped.illumina.mosaik.YRI.exome.20110411.bam, ftp://ftp.1000genomes.ebi.ac.uk/vol1/ftp/phase1/data/NA19257/exome_alignment/NA19257.mapped.illumina.mosaik.YRI.exome.20110521.bam, ftp://ftp.1000genomes.ebi.ac.uk/vol1/ftp/phase1/data/NA12006/exome_alignment/NA12006.mapped.SOLID.bfast.CEU.exome.20110411.bam). The melanoma TCGA mutation data was downloaded from the cBio datahub (https://github.com/cBioPortal/datahub/blob/master/public/skcm_tcga/data_mutations_extended.txt).

## Abbreviations

DAI: Differential Agretopicity Index
DP: Read Depth
GUI: Graphical User Interface
HLA: Human Leukocyte Antigens
NGS: Next Generation Sequencing
RNA-Seq: RNA-Sequencing
SKCM: Skin Cutaneous Melanoma
TCGA: The Cancer Genome Atlas
TPM: Transcripts per Million
